# What is the role of circRNAs in the pathogenesis of cervical cancer? A systematic literature review

**DOI:** 10.3389/fgene.2024.1287869

**Published:** 2024-05-27

**Authors:** Ana Gabrielly de Melo Matos, Gyl Eanes Barros Silva, Eldevan da Silva Barbosa, Marcelo Souza de Andrade, Joyce Santos Lages, Rita da Graça Carvalhal Frazão Corrêa, Ana Gabriela Caldas Oliveira, Eliel Barbosa Teixeira, Marcelli Geisse de Oliveira Prata da Silva, Susanne Suely Santos da Fonseca, Antonio Augusto Lima Teixeira-Júnior, Matheus Silva Alves, Antonio Machado Alencar Junior, André Salim Khayat, Jaqueline Diniz Pinho

**Affiliations:** ^1^ Postgraduate Program in Adult Health, Federal University of Maranhão, São Luís, Brazil; ^2^ Laboratory of Immunofluorescence and Electron Microscopy, University Hospital of the Federal University of Maranhão, São Luís, Brazil; ^3^ Molecular Pathology Study Group, University Hospital of the Federal University of Maranhão, São Luís, Brazil; ^4^ State University of Maranhão, Zé Doca, Maranhão, Brazil; ^5^ University Hospital of the Federal University of Maranhão, São Luís, Brazil; ^6^ Department of Medicine, Federal University of Maranhão, São Luís, Maranhão, Brazil; ^7^ Oncology Research Center, Federal University of Pará, Belém, Pará, Brazil; ^8^ Department of Genetics, Ribeirão Preto Medical School, University of São Paulo, São Paulo, Brazil; ^9^ State University of the Tocantina Region of Maranhão, Department of Health Sciences, Imperatriz, Maranhão, Brazil

**Keywords:** biomarkers, cervical cancer, circRNAs, prognosis, non-coding RNAs

## Abstract

Cervical Cancer (CC) is one of the most prevalent neoplasms among women, considered the leading cause of gynecological death worldwide, and the fourth most common type of cancer. Regional metastasis is closely related to the low effectiveness of treatment, and validating biomarkers can optimize accuracy in diagnosis and prognosis. Among the potential biomarkers associated with disease metastasis are circular RNAs (circRNAs), whose altered expression has been linked to CC progression. In this context, this systematic review aims to compile information on the clinical-pathological significance and describe the biological function of circRNAs. Inclusion and exclusion criteria were used to include relevant literature, followed by *in silico* analysis. Additionally, we employed the UALCAN tools to search for host genes of circRNAs and expression data, miRTargetLink 2.0 to predict interactions of microRNA target genes and the Cytoscape software to predict possible interactions of microRNA target genes. According to the research, most circRNAs were found to be overexpressed and described as regulators of processes such as invasion, cell proliferation, apoptosis and migration. They were also implicated in clinical significance, including metastasis, TNM staging and microRNA interactions. CircRNAs may participate in critical processes in tumorigenesis; therefore, understanding the underlying molecular mechanisms of gene regulation in CC can contribute to the accuracy of diagnosis, prognosis and therapy.

## 1 Introduction

Cervical Cancer (CC) ranks among the most prevalent neoplasms in women, with Human Papillomavirus (HPV) infection as it primary risk factor (Ward et al., 2020). Despite the availability of vaccines, CC remains a significant cause of gynecological mortality globally and stands as the fourth most commonly diagnosed cancer (Sung et al., 2021). Importantly, metastasis constitutes the primary cause of cancer-related deaths in CC patients, leading to adverse prognoses and suboptimal therapeutic outcomes (Hsieh et al., 2021).

The utilization of biopsies, imaging examinations, and biomarkers has been acknowledged as a practical approach to cancer diagnosis (Vaidyanathan et al., 2018). From a clinical perspective, biomarker identification plays a pivotal role, as they serve as tools to evaluate neoplasia risk, facilitate early detection, and enable accurate patient diagnosis and prognosis, enhancing decision-making processes (Sarhadi and Armengol, 2022).

Among the potential biomarkers associated with disease metastasis are circular RNAs (circRNAs), circRNAs represent a class of non-coding RNAs (ncRNAs) generated through a specific type of alternative splicing known as back-splicing. These biomolecules function as competitive endogenous RNAs (ceRNA) binding to microRNAs (miRNAs) and creating a network of post-transcriptional gene regulation (Hosseini et al., 2017). Different combinations of sequences give rise to three categories of circRNAs: exonic circRNAs (EcRNA), intronic circRNAs (CiRNA), and exon-intron circRNAs (EIcRNA) (Anastasiadou et al., 2017).

CircRNAs play pivotal roles in critical processes of tumorigenesis, including cell proliferation, migration and invasion (Hong et al., 2019). Altered expression of circRNAs has been linked to cancer progression, exemplified by SMARCA5, a circRNA contributing to proliferation, invasion, and migration in bladder tumors (Tan et al., 2019), non-small cell lung cancer (Tong, 2020), prostate cancer (Kong et al., 2017), nasopharyngeal cancer (Wang et al., 2023) and CC (Tian et al., 2018). Another noteworthy circRNA is circ-MYBL2, characterized as an oncogene and associated with reduced overall survival in CC patients, making it a potential marker for CC (Wang et al., 2019).

In this context, this systematic review aims to compile information on the clinical-pathological significance and describe the biological function of circRNAs.

## 2 Methodology

### 2.1 Study design and protocol registration

This research is a systematic literature review registered with the International Prospective Register of Systematic Reviews (PROSPERO—https://www.crd.york.ac.uk/PROSPERO/) under registration number CRD42023402481. The study adhered meticulously to the Preferred Reporting Items for Systematic Reviews and Meta-Analyses (PRISMA—https://www.prisma-statement.org/) guidelines. For this systematic review, rigorous inclusion and exclusion criteria were applied, employing specific keywords.

### 2.2 *In silico* analysis

The exploration of host gene of circRNA the tool was used circinteractome (https://circinteractome.nia.nih.gov/index.html) (Dudekula and Panda, 2016) and their expression data was undertaken utilizing the online tool UALCAN (https://ualcan.path.uab.edu/analysis.html) (Chandrashekar et al., 2022), which is linked to TCGA, thus enabling comprehensive data analysis. To forecast plausible interactions of microRNA target genes regulated by the circRNAs identified in this systematic review, miRTargetLink 2.0 (https://ccb-compute.cs.uni-saarland.de/mirtargetlink2) was harnessed. It is worth highlighting that only strongly correlated target genes were considered, validated through techniques such as RT-qPCR, Western blot, cell assays, and/or luciferase reporter assays. Furthermore, Cytoscape (https://cytoscape.org/) was employed to predict potential interactions between microRNAs and target genes (Shannon et al., 2003).

### 2.3 Research question

The crux of this study rests upon the following query: “What are the roles of circular RNAs in the pathogenesis of cervical cancer?” In pursuit of this, the PRISMA-scR protocol was meticulously employed, with the PICOS acronym encompassing: Population—patients who have CC; Intervention - the efficacy of circRNA biomarkers; Comparison—inapplicable; Outcome—the relationship between biomarker expression; Study design—confined solely to experimental studies.

### 2.4 Eligibility criteria

Only original articles reporting experimental studies in English, published between 2018 and 2023, involving patients with anatomopathological diagnoses of CC, were deemed eligible. Moreover, this systematic review encompassed solely works providing tumor tissue samples and presenting clinical data, including TNM staging, metastasis, stage, cell differentiation pattern, and expression levels. Deliberation extended to the implications of circRNAs in proliferation, migration, invasion, cell cycle, apoptosis, participation in epithelial-mesenchymal transition, and whether these ncRNAs target microRNAs. The exclusion criteria encompassed texts presented in abstract, report, review, or monograph formats.

### 2.5 Data sources and strategies

Searches were diligently conducted across electronic databases, including the U.S. National Library of Medicine (PubMed), ScienceDirect and Cochrane. The selected descriptors included: “Circular RNA and Uterine Cervical Neoplasms,” “circRNA and cervical cancer,” and “circRNA and uterine cancer.”

### 2.6 Study selection and strategies

Data selection entailed the removal of duplicates and studies not conforming to the specified inclusion criteria. Information from each article was meticulously organized within a Microsoft Excel 2019 spreadsheet. Each study’s details encompassed clinicopathological characteristics (TNM staging, metastasis, stage, cell differentiation pattern, expression level); implications of circRNAs in proliferation, migration, invasion, cell cycle, apoptosis, involvement in epithelial-mesenchymal transition, whether these ncRNAs target microRNAs, genes and regulate pathways; clinical indicators (diagnostic indicators, prognostic indicators, biomarkers).

### 2.7 Assessment of methodological quality of included studies

The assessment of the methodological quality of the studies included was executed independently by researchers, utilizing the Joanna Institute Critical Appraisal Tools (JBI) checklist (JBI, 2020). Each criterion was meticulously classified as “yes,” “no,” “unclear,” or “not applicable.” The classification of bias risk was assigned based on scores: 1 to 3, “yes,” indicating high bias risk; 4 to 6, “yes,” indicating moderate bias risk; and 7 to 8, indicating low bias risk.

## 3 Results

**FIGURE 1:**
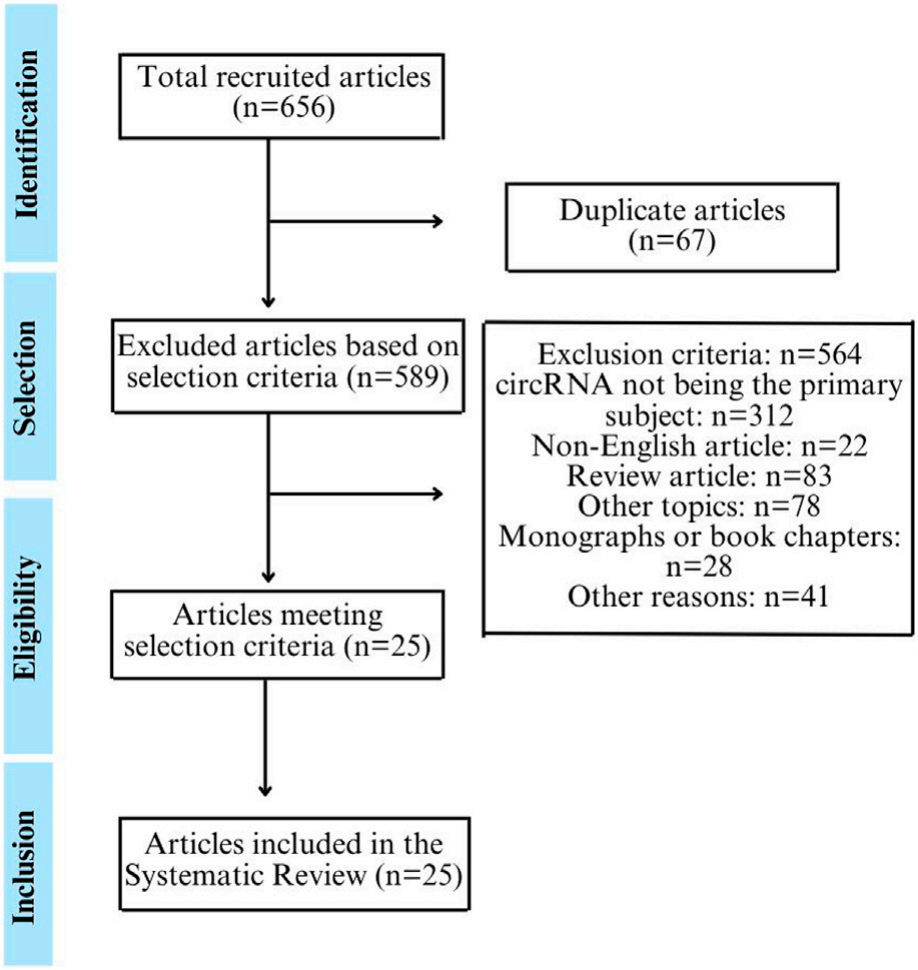
Flowchart depicting the selection and identification of studies, following the methodological steps outlined in the PRISMA guidelines.

Following the specified search strategy and study eligibility criteria, 656 articles were identified across the three databases, following the PRISMA guidelines. After removing duplicates (*n* = 39) and articles that did not meet the selection criteria (*n* = 589), only 25 articles remained for this systematic review, describing the circRNAs associated with CC and examining their potential roles in diagnosis, prognosis and treatment, as depicted in the study selection and identification flowchart following the PRISMA methodology (Figure 1).

### 3.1 Roles of circRNAs in cervical cancer

CircRNAs play pivotal roles in tumor pathogenesis, encompassing proliferation, migration, invasion, metastasis, epithelial-mesenchymal transition (EMT), and cell cycle (Zhang et al., 2021). Through this systematic review, it was observed that the majority of analyzed circRNAs are linked to cell proliferation (*n* = 21), invasion (*n* = 22), migration (*n* = 20), circRNAs acting as circRNAs acting as ceRNA (*n* = 22), and those involved in processes like EMT (*n* = 3) and other pathways in CC (*n* = 3), as detailed in Table 1.

**TABLE 1:** Clinical significance of circRNAs as diagnostic, prognostic and therapeutic indicators.

Circular RNA	Clinical Significance	Diagnostic, Prognostic, and Therapeutic Indicators	Reference
circ_0000730	Correlation with FIGO Stage III–IV (*p* = 0.0489). Reduced overall survival (*p* = 0.0358)	Prognostic biomarker	Yuan et al. (2021)
circ_0043280	Lymph node metastasis (*p* = 0.0006). FIGO Stage II (*p* = 0.024). Reduced disease-free survival (DFS) (*p* = 0.0015) and decreased overall survival (OS) (*p* < 0.001); Tumor size > 4 cm (*p* = 0.0007)	Diagnostic and therapeutic biomarkers	Zhang et al. (2021)
circ_0011385	Correlation with FIGO Stage II (*p* = 0.024)	Prognostic biomarker	Xu et al. (2021)
circRNA_101996	Lymph node metastasis (*p* = 0.038). Overall survival (OS) *p* = 0.020, FIGO Stage III–IV (*p* = 0.004), tumor size > 4 cm (*p* = 0.020)	Prognostic and diagnostic biomarkers	Song et al. (2021)
circCDKN2B-AS1	Lymph node metastasis (*p* = 0.044). FIGO Stage IIA (*p* = 0.022), tumor size > 4 cm (*p* = 0.029)	Diagnostic biomarker	Zhang et al. (2020)
circ_0007364	Lymph node metastasis (*p* < 0.05). FIGO Stage IV (*p* < 0.05). Overall survival (OS) *p* = 2.8e-05	Prognostic and diagnostic biomarkers	Chen et al. (2020)
circ_0018289	Lymph node metastasis (*p* = 0.005). FIGO Stage IIA (*p* = 0.005), tumor size > 4 cm (*p* = 0.009), sensitivity 0.9. Disease-free survival (DFS) *p* = 0.005 and overall survival (OS) *p* = 0.015	Disease monitoring and prognostic biomarker	He et al. (2020)
circAGFG1	FIGO Stage III-IV (*p* = 0.004), Disease-Free Survival *p* < 0.05, tumor size > 4 cm (*p* < 0.05)	Diagnostic biomarker	Wang et al. (2020)
circCLK3	Metastasis. FIGO Stage II (*p* = 0.002), tumor differentiation G3 (*p* = 0.015). Worse Disease-Free Survival (DFS) *p* < 0.01 and Overall Survival (OS) *p* < 0.05	Diagnostic biomarker and therapeutic target.	Hong et al. (2019)
circ_0075341	Lymph node metastasis *p* < 0.05. FIGO Stage IV (*p* < 0.05). Poorer Overall Survival (OS) *p* = 0.02. Tumor size > 4 cm (*p* < 0.05)	Diagnostic biomarker	Shao et al. (2020)
circ_0005576	Lymph node metastasis *p* < 0.05. FIGO Stages IIA-IIB. Overall Survival (OS) *p* < 0.01	Diagnostic biomarker	Ma et al. (2019)
circEIF4G2	Lymph node metastasis (*p* = 0.011). Overall Survival *p* < 0.05. Tumor size > 4 cm (*p* = 0.003)	Diagnostic biomarker	Mao et al. (2020)
circRNA8924	FIGO Stages IIA-IIB (*p* = 0.041), Tumor size > 4 cm (*p* = 0.008)	Diagnostic biomarker	Liu et al. (2018)
circ_0067934	Lymph node metastasis (*p* < 0.05). FIGO Stage II (*p* < 0.05). Overall Survival (OS) *p* < 0.05	Diagnostic biomarker	Hu et al. (2019)
circ-ATP8A2	Lymph node metastasis (*p* = 0.009), positive lymph node invasion, myometrial invasion, poor prognosis. FIGO Stages IIA-IIB (*p* = 0.026). Overall Survival (OS) *p* = 0.007	Diagnostic biomarker	Ding and Zhang. (2019)
circ_0001038	Lymph node metastasis (*p* = .0014). Overall Survival *p* = 0.03	Prognostic and diagnostic biomarkers	Wang et al. (2020)
circRNA_101996	Lymph node metastasis (*p* = 0.010). FIGO Stages III–IV (*p* = 0.020), significantly worse Overall Survival (OS) *p* = 0.032	Diagnostic biomarker	Song et al. (2019)
circ0001955	FIGO Stages IA2 to IIA2, metastasis	Prognostic and diagnostic biomarkers	Wang et al. (2023)
circ_0000388	Lymph node metastasis (*p* = 0.004)	Diagnostic biomarker	Meng et al. (2021)
circ_0087429	Lymph node metastasis (*p* = 0.031). FIGO Stages IIA-IIB (*p* = 0.042)	Therapeutic target.	Yang et al. (2022)
circ-E2F3	Lymph node metastasis (*p* < 0.0001), FIGO Stages IIA-IIB (*p* < 0.0001), tumor size > 4 cm (*p* < 0.0001)	Diagnostic biomarker	Cao et al. (2022)
circSAMD11	Overall Survival (OS) (*p* < 0.05)	Diagnostic biomarker	Pan et al. (2022)
circFAT1	Lymph node metastasis (*p* < 0.05), FIGO Stage III (*p* < 0.005). Overall Survival (OS) *p* < 0.05	Diagnostic biomarker	Zhou et al. (2021)
circSOS2	FIGO Stage III (*p* = 0.010). Poorer Overall Survival (OS) *p* = 0.001, tumor size > 4 cm (*p* = 0.010)	Diagnostic biomarker	Li et al. (2021)
circ_0109046	Lymph node metastasis (*p* < 0.05), FIGO Stages III-IV (*p* < 0.01), Worse Overall Survival (OS) *p* < 0.05	Diagnostic biomarker	Li et al. (2021)

These ncRNAs exhibit differential expression between cancerous and healthy tissues (Salzman et al., 2013). Of the 25 circRNAs included in this study, 23 acted as oncogenes, while 02 acted as tumor suppressors, namely, circ_0000730 (Yuan et al., 2021) and circ_0087429 (YANG et al., 2022), as observed in Table 1.

In terms of clinical significance, circRNAs were associated with higher Federation of Gynecology and Obstetrics (FIGO) Stage (*n* = 22) and lymph node metastasis (*n* = 19) and linked to decreased survival, as seen in Table 1. Furthermore, based on this investigation, circRNAs were described as diagnostic biomarkers (*n* = 18), prognostic indicators (*n* = 3) and therapeutic targets (*n* = 6) (Table 1).

### 3.2 *In silico* analysis

**FIGURE 2:**
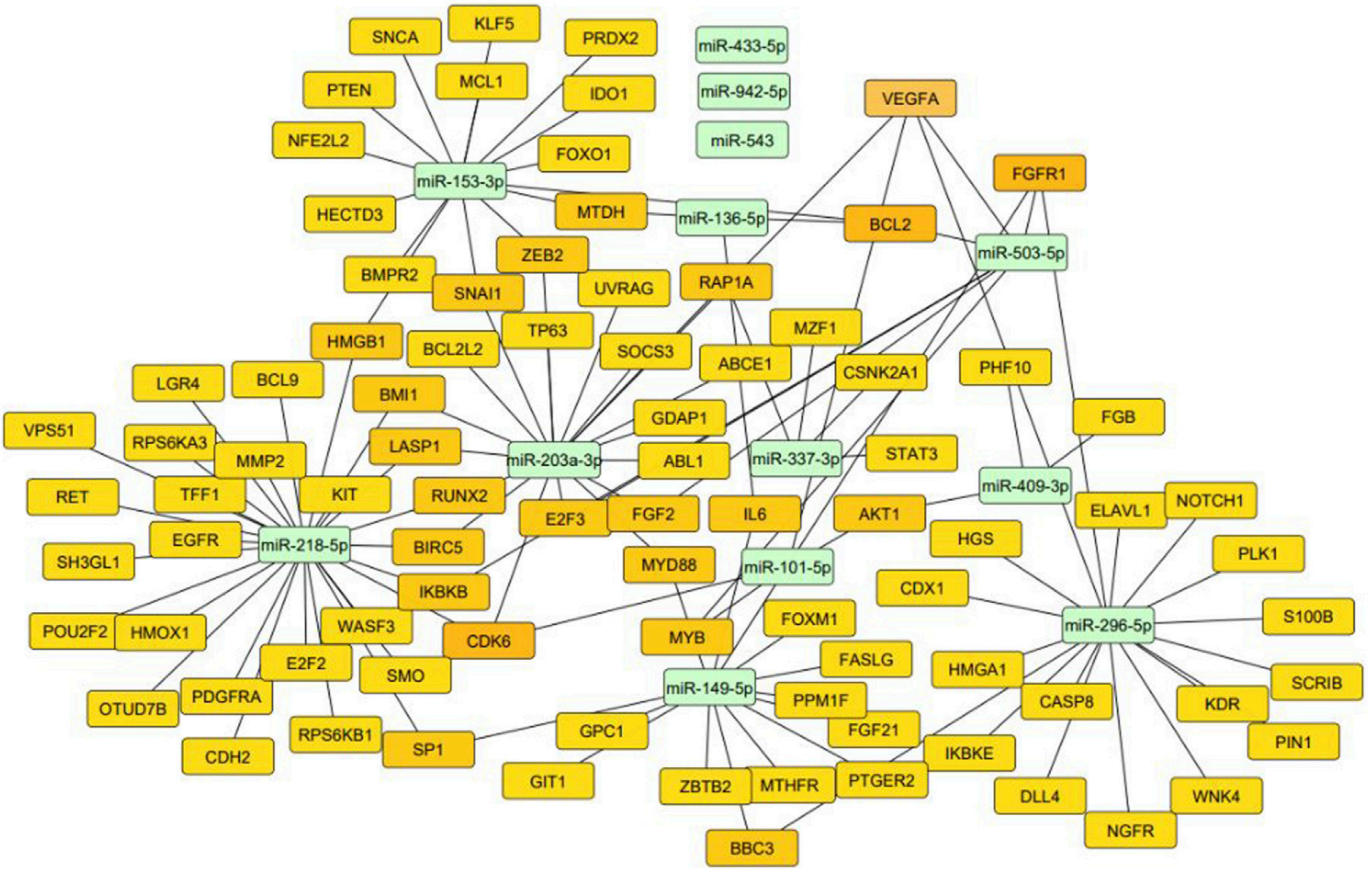
miRNA-Target Gene Interaction. Source: Cytoscape.

The predicted target genes of these miRNAs were explored using the online platform miRTargetLink 2.0, yielding 2,527 target genes regulated by 17 miRNAs. After excluding weakly correlated target genes, only 208 target genes remained, regulated by miRNAs. Notably, three miRNAs lacked corresponding target genes among the analyzed miRNAs, as observed in Figure 2.

## 4 Discussion

### 4.1 CircRNAs involved in proliferation, invasion, migration, angiogenesis and microRNA sponges

CircRNAs play a significant role in the development of CC, as they can influence various processes related to tumor pathogenesis, such as proliferation, EMT, migration, invasion and angiogenesis. These characteristics can converge to trigger factors of worse prognosis (Yin et al., 2020; Zhang et al., 2021).

Among the articles included in this review that report the biological function of circRNAs in CC, the work of Zhang et al. (2020) stands out, which pointed out that the aberrant expression of circCDZN2B-AS1 induced the malignant phenotype *in vivo* and *in vitro*. Furthermore, in the cited study, it was also observed that this circRNA is correlated with CC progression and cell metabolic activity, interacting with the IMP3 protein. The IMP3 protein plays an essential role in cancer progression by acting as an mRNA stabilizer for the *MEKK1* gene, activating the *MEK1/ERK* signaling pathway, and promoting cell growth and proliferation (Zhang et al., 2021).

The circ_0007364 was associated with the progression of this tumor type, mainly with cell proliferation, by regulating the expression of the *MAT2A* gene, with the underlying regulatory mechanism related to the inhibition of miRNA-101-5p, known as a tumor suppressor (Chen et al., 2020). In another study, increased expression of the *MAT2A* gene was related to cell growth in CC under glucose deprivation conditions, significantly correlating with poor prognosis and advanced stages of patients (Luo et al., 2022).

The role of these biomolecules as ceRNAs of miRNAs should also be considered. CeRNAs can inhibit miRNA expression, reducing miRNA-mRNA interaction (Wang et al., 2021). For example, the study by Hong et al. (2019) suggests that circCLK3 is a potential diagnostic biomarker, promoting proliferation, migration, EMT, and invasion, acting as a ceRNA for miR-320a by regulating the *FoxM1* gene. Therefore, the circCLK3/miR-320a/FoxM1 axis may play a relevant role in CC progression. In another study, circRNA_101996 acted as a ceRNA, negatively regulating miR-1236-3p and inhibiting the expression of the tumor suppressor *TRIM37*, resulting in CC proliferation and progression (Song et al., 2021).

Additionally, circRNAs may be involved in regulating EMT and other vital pathways. EMT is a process by which neoplastic epithelial cells change their phenotypic characteristics, acquiring characteristics of mesenchymal cells. During EMT, epithelial cells lose adherence to each other and the extracellular matrix and gain mobility and invasive capacity (Pastushenko and Blanpain, 2019). Zhou et al. (2020) suggest that circFAT1 may activate the *ERK1/2* signaling pathway (Extracellular Signal-Regulated Kinase 1/2) through negative regulation of the tumor suppressor miR-409-3p, thus inducing proliferation, migration, and cell invasion, all of which are related to CC progression. In another study, *ERK1/2* pathway activation was responsible for cell cycle progression in HK2-modified cells, recognized as a critical regulator in malignant growth in various cancers (Cui et al., 2020).

A deeper understanding of all these biological functions may reveal potential therapeutic targets, such as circRNA_101996 (Song et al., 2021), circCLK3 (Hong et al., 2019), and circ_0001038 (Wang and Li, 2020), as observed in Table 1.

### 4.2 CircRNAs as potential diagnostic and prognostic biomarkers: differential expression in advanced tumor stages

The utilization of circRNAs as biomarkers holds significant promise across various diseases, owing to their unique attributes, including stability, resistance to degradation, sensitivity, precision and tissue-specific expression, which enable precise regulation of gene expression (Zhou et al., 2020). These molecules have attracted interest as biomarkers because their circular structure renders them highly resistant to exonuclease degradation, resulting in prolonged half-lives and remarkable stability, especially in cancer contexts. Moreover, their broad expression in human tissue samples further enhances their biomarker potential (Li et al., 2015; Enuka et al., 2016).

He et al. (2020) presented circRNA_0018289 as a potential biomarker. In this study, the authors analyzed 192 tumor samples and adjacent tissues, where the overexpression of this circRNA was associated with lymph node metastasis, reduced disease-free survival, and tumor size ≥4 cm. Additionally, with an AUC curve of 0.9, sensitivity (80.7%), and specificity (89.6%), this circRNA can differentiate between tumor and adjacent non-tumor tissues.

Another notable candidate, circAGFG1, was implicated in CC progression due to its suppressing *p53*, a pivotal tumor suppressor and regulator of cellular stress response. Mutations in the *p53* gene can disrupt its tumor-suppressive function, contributing to the proliferation of abnormal cervical cells (Wang et al., 2020). In the study, circAGFG1’s overexpression correlated with higher tumor recurrence rates, extensive tumor invasion, and diminished overall patient survival.

These findings yield valuable insights into disease severity and CC progression. The identification of robust biomarkers is pivotal for early and accurate diagnosis. Current literature underscores the expansive clinical potential of RNA-based biomarkers, particularly their stable expression in bodily fluids, which provides efficient prognostic information for the perioperative period, often surpassing conventional clinical parameters such as tumor size and clinical-pathological stage (Ding and Zhang, 2019).

### 4.3 CircRNAs potential ferramenta in therapy: circRNA_101996 and circ_0007364

Research focused on exploring circRNAs for therapeutic purposes mainly utilizes RNA interference (siRNA) or antisense oligonucleotide (ASO) techniques (Zhang et al., 2021). Among these strategies, using siRNAs to trigger the degradation or reduction of circRNA expression is the most commonly employed therapeutic approach (Hsiao et al., 2017).

In a study by Song et al. (2021), the knockdown technique involving shRNA (a type of RNA interference) was employed to reduce the expression of circRNA_101996 significantly. This reduction led to a substantial decrease in cell proliferation, migration and invasion within neoplastic cells. These findings underscore the promising potential of inhibiting circRNA_101996 as a viable therapeutic strategy for treating CC (Table 2)

**TABLE 2:** List of studies on circRNAs presenting sample type, host gene, target miRNA and biological function.

Circular RNA	Sample type	Host gene	Target miRNA	Biological function	Reference
circ_0000730 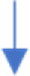	*50 samples of cancerous and adjacent normal tissue	PITPNA 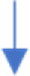	miR-942-5p 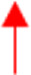	Inhibited *in vitro* proliferation, invasion, and trans-endothelial migration	Yuan et al. (2021)
circ_0043280 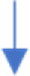	140 samples of cancerous and adjacent normal tissue	N/A***	miR-203a-3p 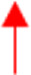	inhibit tumor growth and metastasis	Zhang et al. (2021)
circ_0011385 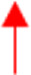	**50 samples of cancerous and adjacent normal tissue	EIF3I 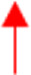	miR-149-5p	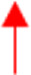 Cellular proliferation, migration, and invasion. Activates the MAPK signaling pathway	Xu et al. (2021)
circRNA_101996 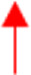	60 samples of cancerous and adjacent normal tissue	N/A	miR-1236-3p 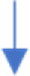	Cellular proliferation, migration, and invasion	Song et al. (2021)
circCDKN2B-AS1 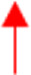	46 samples of normal cervical epithelial tissue, 41 samples of high-grade squamous intraepithelial lesions, and 75 samples of CC tissue	N/A	N/A	Cellular proliferation, migration, and invasion. Correlation with HK2 enzyme (a limiting enzyme in the aerobic glycolysis pathway)	Zhang et al. (2020)
circ_0007364 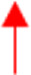	53 samples of cancerous and adjacent normal tissue	PTP4A2 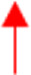	miR-101-5p 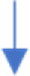	Cellular proliferation and invasion	Chen et al. (2020)
circ_0018289 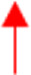	192 samples of cancerous and adjacent normal tissue	SYT15 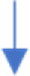	N/A	Cellular proliferation, migration, and invasion	He et al. (2020)
circAGFG1 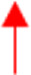	39 samples of cancerous tissues and adjacent normal tissues	N/A	N/A	Cellular proliferation, migration, and invasion suppress p53	Wang et al. (2020)
circCLK3 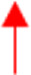	48 samples of cancerous tissues and adjacent normal tissues	N/A	miR-320a 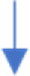	Cell proliferation, migration, stromal invasion, and EMT.	Hong et al. (2019)
circ_0075341 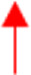	37 samples of cancerous tissues and adjacent normal tissues	MAPK9 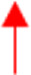	miR-149-5p 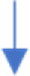	Cell proliferation and invasion	Shao et al. (2020)
circ_0005576 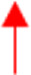	68 samples of cancerous and adjacent normal tissues	CDC42	miR-153-3p 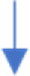	Proliferation, migration, and invasion	Ma et al. (2019)
circEIF4G2 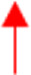	20 samples of cancerous and adjacent normal tissues	N/A	miR-218 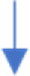	Cell proliferation and migration	Mao et al. (2020)
CircRNA8924 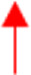	33 samples of cancerous and adjacent normal tissues	N/A	miR-519a-5p 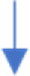	Cell proliferation, migration, and invasion	Liu et al. (2018)
circ_0067934 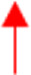	61 samples of cancerous tissues and 21 samples of adjacent normal tissues	PRKCI 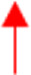	miR-545 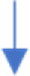	Cell proliferation, colony formation, migration, invasion, and EMT	Hu et al. (2019)
Circ-ATP8A2 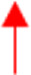	46 samples of cancerous and adjacent normal tissues	N/A	miR-433 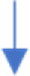	Myometrial invasion	Ding and Zhang. (2019)
circ_0001038 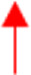	55 samples of cancerous and adjacent normal tissues	POLR1A	miR-337-3p 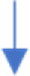	Cellular proliferation, migration, and invasion	Wang et al. (2020)
circ0001955 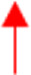	15 samples of cancerous and adjacent normal tissues	N/A	miR-188 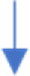	Proliferation, invasion, and migration	Wang and Ren. (2023)
circRNA_101996 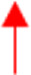	39 samples of cancerous and adjacent normal tissues	N/A	miR-8075 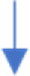	Cellular proliferation, migration, and invasion	Song et al. (2019)
circ_0000388 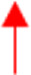	40 samples of cancerous and adjacent normal tissues	N/A	miR-337-3p 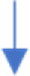	Cellular proliferation, migration, depth, and invasion	Meng et al. (2021)
circ_0087429 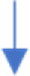	44 samples of cancerous and adjacent normal tissues	SPIN1	miR-5003-3p 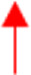	Inhibits migration, invasion, and angiogenesis	Yang et al. (2022)
circ-E2F3 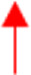	63 samples of cancerous and adjacent normal tissues	N/A	miR-296-5p 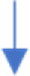	Proliferation, invasion, and migration. Increases Cyclin D1 expression	Cao et al. (2022)
circSAMD11 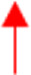	62 samples of cancerous and adjacent normal tissues	N/A	miR-503 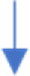	Proliferation, invasion, and migration. Regulates the Wnt/β-catenin pathway	Pan et al. (2022)
circFAT1 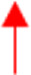	47 samples of cancerous and adjacent normal tissues	N/A	miR-409-3p 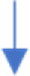	Activates the ERK1/2 pathway	Zhou et al. (2021)
circSOS2 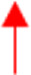	53 samples of cancerous and adjacent normal tissues	ZNF700 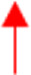	miR-543 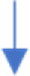	Proliferation, invasion, and migration	Li et al. (2021)
circ_0109046 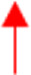	50 samples of cancerous and adjacent normal tissues	N/A	miRNA-105 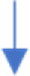	Proliferation, invasion, and migration	Li et al. (2021)

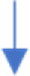
* = Downregulated expression.

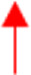
** = Upregulated expression.

***N/A, not available.

****Host gene= Research conducted using circinteractome tools and expression data using UALCAN.

In a separate study, Chen et al. (2020) utilized shRNA to suppress the expression of circ_0007364. This suppression reduced the expression of the methionine adenosyltransferase II alpha (*MAT2A*) gene. Consequently, this suppression could impede cellular processes that play a role in CC progression.

RNA interference-based therapies hold intriguing possibilities and challenges within cancer treatment. They address the primary drivers of tumorigenesis, offering a unique ability to selectively modulate mRNA expression of genes critically involved in carcinogenesis (Cuciniello et al., 2021).

### 4.4 *In silico* analysis

The *in silico* analysis uncovered that one of the genes regulated by miRNAs and targeted by circRNAs governs the Vascular Endothelial Growth Factor (*VEGF*), as illustrated in Figure 2. VEGF holds the distinction of being a principal regulator of angiogenesis in disease development. Its influence extends to controlling both the physiological and pathological growth of blood vessels (Melly and Banfi, 2022).

The inhibition of *VEGF* serves as the foundation for anti-angiogenic therapies, which have been extensively studied in various conditions, including cancer. These strategies involve the application of monoclonal antibodies to block *VEGF*, effectively restraining the pathological angiogenesis occurring within the tumor microenvironment and thereby limiting neoplastic growth (Tan, 2019). In the study conducted by Zhang et al. (2018) and Guo et al. (2019), it was observed that circ_0023404 and *VEGF* were upregulated in CC. These elements positively regulated metastasis and chemoresistance through the miR-5047/VEGF pathway.

Extrinsic factors, encompassing the expression of angiogenic markers such as *VEGF* and *HIF-1α*, can impact resistance to chemoradiotherapy and tumor (Zhang and Brekken, 2022).

A more profound understanding of the regulatory mechanisms of circRNAs associated with enhanced resistance to chemotherapy could potentially unveil therapeutic targets that enhance treatment efficacy (Zhang et al., 2020).

## 5 Study limitations

This literature review maintains a focused approach, documenting marker types, research methodologies, action mechanisms, functions, expressions, sample sizes, and other circRNA indicators. Nevertheless, the study has its inherent limitations. Primarily, the analysis predominantly occurred *in silico*, curtailing new biomarkers’ tangible validation. Despite the significance of the employed bioinformatics tools, laboratory investigations are required to substantiate the findings. Additionally, due to the scarcity of literature, a comprehensive explication of every biological process governed by circRNAs was unattainable. Lastly, our scope encompassed only circRNAs associated with diagnostic, therapeutic and prognostic markers. Regarding the information on the association between expression. HPV and circRNA, among the articles included, only one circRNA (CircCDKN2B-AS1) exhibited overexpression in HPV16-positive cervical cancer, demonstrating statistical significance (Zhang et al., 2020).

Furthermore, only one study provided sensitivity and specificity data: He et al. (2020), which investigated circ_0018289. The circRNAs included in our analysis may only comprehensively represent part of the spectrum of circRNA studies. Nevertheless, our analysis thoroughly encompasses a significant portion of circRNAs as biomarkers in CC, sufficiently portraying the present state of circRNA research as markers for CC over the past 5 years.

## 6 Conclusion

In CC, the literature underscores the involvement of circRNAs in several stages of carcinogenesis, correlating with cell proliferation, migration and invasion. Moreover, they are associated with adverse prognostic factors, highlighting their potential for future applications, whether through non-invasive sample utilization or therapy, particularly those exhibiting significant specificity and sensitivity, such as circRNA_0018289. Despite this potential, only a limited number of studies delve into their investigation and potential utility, underscoring the necessity for further research to comprehensively understand the regulatory and functional roles of circRNAs in CC.

## Data Availability

The original contributions presented in the study are included in the article/Supplementary Material, further inquiries can be directed to the corresponding author.
