# Supplementary material for: What is the role of circRNAs in the pathogenesis of cervical cancer? A systematic literature review
Source: Front Genet. 2024 May 27;15:1287869. doi: 10.3389/fgene.2024.1287869 (PMC11163134; doi:10.3389/fgene.2024.1287869)
Supplement: Supplementary file 1 [file Table1.DOCX]

***Supplementary Material***

# Supplementary Figures and Tables

## Supplementary Tables

**Supplementary Table 1.** Evaluation of the quality of methods utilized in the included studies using the *Joanna Institute Critical Appraisal Tools* (JBI).

| **Reference** | **Q1** | **Q2** | **Q3** | **Q4** | **Q5** | **Q6** | **Q7** | **Q8** | **Q9** | **Total**  **(YES)** | **Trend level** |
| --- | --- | --- | --- | --- | --- | --- | --- | --- | --- | --- | --- |
| Yuan et al., (2021) | YES | YES | N / D | YES | YES | SIM | *N / D | YES | YES | 7 | Low |
| Zhang et al., (2021) | YES | YES | N / D | YES | YES | N / D | YES | YES | YES | 7 | Low |
| Xu et al., (2021) | YES | YES | N / D | YES | N / D | YES | N / D | YES | N / D | 5 | Moderate |
| Song et al., (2021) | YES | YES | N / D | YES | YES | YES | N / D | YES | YES | 7 | Low |
| Zhang et al., (2020) | YES | YES | N / D | YES | N / D | YES | N / D | YES | YES | 6 | Moderate |
| Chen et al., (2020) | YES | YES | N / D | YES | N / D | YES | N / D | YES | YES | 6 | Moderate |
| He et al., (2020) | YES | YES | N / D | YES | N / D | YES | YES | YES | YES | 7 | Low |
| Wang et al., (2020) | YES | YES | N / D | YES | N / D | YES | YES | YES | YES | 7 | Low |
| Hong et al., (2019) | YES | YES | N / D | YES | N / D | U** | N / D | YES | YES | 5 | Moderate |
| Shao et al., (2020) | YES | YES | N / D | YES | YES | N / D | N / D | YES | YES | 7 | Low |
| Ma et al., (2019) | YES | YES | N / D | YES | N / D | YES | YES | YES | YES | 7 | Low |
| Mao et al., (2019) | YES | YES | N / D | YES | N / D | YES | YES | YES | YES | 7 | Low |
| Liu et al., (2019) | YES | YES | N / D | YES | N / D | YES | YES | YES | YES | 7 | Low |
| Hu et al., (2019) | YES | YES | N / D | YES | N / D | YES | YES | YES | YES | 7 | Low |
| Ding et al., (2017) | YES | YES | N / D | YES | N / D | YES | YES | YES | YES | 7 | Low |
| Wang et al., (2020) | YES | YES | N / D | YES | N / D | YES | YES | YES | YES | 7 | Low |
| Song et al., (2019) | YES | YES | N / D | YES | N / D | YES | YES | YES | YES | 7 | Low |
| Meng et al., (2020) | YES | YES | N / D | YES | N / D | YES | YES | YES | YES | 7 | Low |
| Yang et al., (2022) | YES | YES | N / D | YES | N / D | YES | N / D | YES | YES | 6 | Moderate |
| Cao et al., (2021) | YES | YES | N / D | YES | N / D | YES | YES | YES | YES | 7 | Low |
| Pan et al., (2021) | YES | YES | N / D | YES | N / D | YES | N / D | YES | N / D | 5 | Moderate |
| Xu et al., (2021) | YES | YES | N / D | YES | N / D | YES | YES | YES | YES | 7 | Low |
| Zhou et al., (2020) | YES | YES | N / D | YES | N / D | YES | N / D | YES | YES | 6 | Moderate |
| Li et al., (2021) | YES | YES | N / D | YES | N / D | YES | YES | YES | YES | 7 | Low |
| Zhang et al., (2018) | YES | YES | N / D | YES | N / D | YES | YES | YES | YES | 7 | Low |

*NA= Not Applicable

**U= Uncertain
